# Carvacrol Alleviates Prostate Cancer Cell Proliferation, Migration, and Invasion through Regulation of PI3K/Akt and MAPK Signaling Pathways

**DOI:** 10.1155/2016/1469693

**Published:** 2016-10-10

**Authors:** Yun Luo, Jie-Ying Wu, Min-Hua Lu, Zhi Shi, Ning Na, Jin-Ming Di

**Affiliations:** ^1^Department of Urology, The 3rd Affiliated Hospital of Sun Yat-Sen University, Guangzhou, Guangdong 510630, China; ^2^Department of Cell Biology & Institute of Biomedicine, National Engineering Research Center of Genetic Medicine, Guangdong Provincial Key Laboratory of Bioengineering Medicine, College of Life Science and Technology, Jinan University, Guangzhou, Guangdong 510632, China; ^3^Department of Kidney Transplantation, The 3rd Affiliated Hospital of Sun Yat-Sen University, Guangzhou, Guangdong 510630, China

## Abstract

TRPM7 is a potential therapeutic target for treatment of prostate cancer. In this study, we investigated the effects of nonselective TRPM7 inhibitor carvacrol on cell proliferation, migration, and invasion of prostate cancer PC-3 and DU145 cells. Our results showed that carvacrol blocked TRPM7-like currents in PC-3 and DU145 cells and reduced their proliferation, migration, and invasion. Moreover, carvacrol treatment significantly decreased MMP-2, p-Akt, and p-ERK1/2 protein expression and inhibited F-actin reorganization. Furthermore, consistently, TRPM7 knockdown reduced prostate cancer cell proliferation, migration, and invasion as well. Our study suggests that carvacrol may have therapeutic potential for the treatment of prostate cancer through its inhibition of TRPM7 channels and suppression of PI3K/Akt and MAPK signaling pathways.

## 1. Introduction

Prostate cancer (PCa) is the second leading cause of cancer-related death in men [1–3]. Although multiple treatment options are available, it is currently lack of effective therapies for the treatment of androgen-independent prostate cancer which often arises after hormonal deprivation or ablation therapy [4].

Transient receptor potential melastatin-like 7 channel (TRPM7) is a member of melastatin-like transient receptor potential (TRPM) subfamilies, widely expressed in mammalian cells [5]. It is permeable to Ca^2+^ and Mg^2+^ and other divalent cations and has an alpha-kinase domain [6]. It is found that TRPM7 is highly expressed in a number of human cancer tissues and cell lines to regulate cell proliferation, migration, and invasion, such as glioblastoma [7], ovarian cancer [8], and breast cancer [9]. Increasing Ca^2+^ and Mg^2+^ influx promotes the proliferation of prostate cancer cells through activating TRPM7 [10]. Moreover, cholesterol activates TRPM7 and thus increases Ca^2+^ entry, regulating proliferation, migration, and viability of human prostate cells [11]. Inhibition of TRPM7 enhances TNF-related apoptosis inducing-ligand- (TRAIL-) induced apoptosis in PC-3 cells [12], indicating that TRPM7 contributes to the pathogenesis of prostate cancer and serves as a potential therapeutic target for prostate cancer [13]. So far, several signaling pathways were reported to be regulated by TRPM7, including signal Transducer and Activator of Transcription 3 (STAT3), Notch, PI3K/Akt, and MAPK signaling pathways [14, 15]. In prostate cancer cells, knockdown TRPM7 by shRNA inhibited cholesterol-induced Akt or ERK phosphorylation [11]. Hence, it suggests that both PI3K/Akt and MAPK signaling pathways are the downstream mechanisms of TRPM7 functions in prostate cancer.

Carvacrol (CAR) is a natural-bioactive monoterpenoid phenol with multiple uses. It is used as flavor agent in cosmetic and food products and the most active constituent of thyme EOs extracted from many plants, including fruits, vegetables, spices, and herbs. Carvacrol also exhibits antifungal, antiviral, antitumor, and anti-inflammatory activities [16]. Carvacrol was first reported by Parnas et al. as a nonselective TRPM7 inhibitor [17]. The inhibitory effects of carvacrol on TRPM7 and TRPM7-like currents in HEK293 cells and glioblastoma cell line were further confirmed [7]. However, the pharmacological effects of carvacrol on the proliferation, migration, and invasion of prostate cancer cells have not yet been investigated.

In this study, we compared the TRPM7 protein expression between control prostate cells and PCa cells. We further evaluated the effects of carvacrol on TRPM7-like currents, proliferation, migration, and invasion in PC-3 and DU145 cells and investigated the potential underlying mechanisms involved in these effects.

## 2. Materials and Methods

### 2.1. Cell Culture and Reagents

Nonneoplastic human prostatic epithelial cells (RWPE-1) using as control prostate cell line as well as prostate cancer cell lines DU145 (HTB-81) and PC-3 (CRL1435) were obtained from the American Type Culture Collection (Manassas, VA). PWPE-1 cells were maintained in defined keratinocyte serum-free medium (K-SFM) containing 50 *μ*g/mL bovine pituitary extract and 5 ng/mL EGF (Invitrogen, USA). DU145 and PC-3 cells were cultured in DMEM with 10% fetal bovine serum, penicillin (100 U/mL), and streptomycin (100 ng/mL) and maintained at 37°C with 95% humidified air and 5% CO_2_ and passaged as needed. Culture medium was changed twice weekly. Cell culture related materials were purchased from Gibco Life Technologies Corporation (USA). All other reagents used were purchased from Sigma-Aldrich (USA) unless mentioned otherwise.

### 2.2. RNAi Assay

Lentivirus plasmids were obtained from Addgene (Cambridge, MA) in pLKO.1 cloning vector and contained either nonspecific control shRNA (sh-Control) or shRNA specific for human TRPM7 (GeneBank: AY032950). According to the other study, the sequences for TRPM7 and control shRNA were as follows: GTCTTGCCATGAAATACTC and TGTGCTCCGAACGTGTAGT [18]. ShRNA viruses were packaged and produced following the protocol provided online by Addgene Company (http://www.addgene.org/tools/protocols/plko/). PC-3 cells were infected with either lentiviral-sh-Control or lentiviral-sh-TRPM7 (MOI = 40). Culture medium was changed to the fresh medium 24 h after infection. TRPM7, p-Akt, and p-ERK1/2 protein expression were determined at 72 h after infection, and TRPM7-like current was determined as well. In the meantime, cells with infection for 72 h were digested and seeded into the corresponding culture plate to carry out CCK-8 assay, wound healing, and Transwell assay.

### 2.3. CCK-8 Assay

The viability and proliferation of PC-3 and DU145 cells were examined using CCK-8 kit (Tongren Shanghai Co., China) according to the manufacturer's instructions. Briefly, cells were seeded on 96-well plates at a density of 0.5 × 10^5^ cells/well and grown for additional 24 h prior to the experiment. Cells were treated as indicated concentration of carvacrol and corresponding vehicle for 24, 48, and 72 h. Then CCK-8 solution (10 *μ*L) was added to each well and incubated for additional 1 h. Absorbance at 450 nm was measured using a microplate reader (Syngery H1, Biotek, USA). Cell viability was expressed as a percentage of the vehicle control.

### 2.4. Colony Formation

Colony formation experiments were carried out according to our previous study [19, 20]. PC-3 and DU145 cells (300 cells/well) were seeded in 6-well plates overnight and subsequently treated with carvacrol (500 *μ*M) for 24 h, and then it was replaced with fresh culture medium without carvacrol. After that, culture medium was changed every 5 days. After 10 days of culture, cells were fixed with 100% ice-cold Methanol for 10 minutes and stained with 0.5% crystal violet solution for 10 min, then washed with water, and air-dried. Cell colonies images were captured using a digital camera connected to a phase-contrast Olympus microscope (×10 objectives). Colony numbers (containing >50 cells) were determined using Image-Pro Plus software. Data were presented as a percentage of vehicle control.

### 2.5. Wound Healing

Wound healing experiments were carried out according to our previous study [21, 22]. Briefly, cells were seeded in 6-well plates (5 × 10^5^/well) and grown to about 80% confluence, and then the monolayer of cells was scratched with a 200 *μ*L pipette tip to create a wound gap and treated with either carvacrol (500 *μ*M) or corresponding vehicle control for 24 and 48 h. Cells were allowed to migrate in serum-free medium as indicated time point. Cell images and the scratches were photographed using a phase-contrast Olympus microscope (10x objective). Throughout experiments, the same visual field was used. The gap lengths were measured by Image-Pro Plus software.

### 2.6. Transwell Assay

Invasion experiments were carried out according to our previous study [14, 23]. BioCoat Matrigel invasion chambers (8 *μ*m polycarbonate Nucleopore filters, Cat. 354480) were used. Briefly, PC-3/DU145 cells were treated with carvacrol (500 *μ*M) or equivalent vehicle for 24 h. Then 100 *μ*L of cells (2.5 × 10^4^ cells/mL) in FBS-free DMEM was plated in the upper chamber and the lower chamber contained medium with 10% FBS/DEEM. After incubating for 24 h at 37°C in 5% CO_2_, nonmigrated cells in upper chamber were scraped from the upper surface of the membrane using cotton swab. Migrated cells remaining on the bottom surface were fixed with 75% ethanol and stained with crystal violet (0.1%). Finally, images of the invaded cells were photographed and invading cells were counted using Image-Pro Plus software.

### 2.7. Immunofluorescent Staining

Immunofluorescent staining experiments were carried out according to our previous study [24, 25]. Cells were fixed with 4% paraformaldehyde for 30 min at room temperature (RT) and then permeabilized for 30 min with 0.1% Triton X-100 in PBS. Rhodamine phalloidin staining was performed following the manufacturer's instructions. Cells were incubated with rhodamine phalloidin (1 : 50; Molecular Probes, USA) to label F-actin and with DAPI (1 *μ*g/mL, Sigma-Aldrich, USA) to label nucleic acid, for 20 min at RT. Immunofluorescent images were captured from at least 6 randomly chosen areas using Zeiss confocal microscope.

### 2.8. Patch Clamp Recording

Patch clamp experiments were carried out according to Sun et al.'s report [10]. Whole cell currents were recorded using an Axopatch 200B (Axon Instruments, Inc.), with holding potential of 0 mV, 100 ms voltage ramps ranging from −100 to +100 mV, and 2-s intervals at 2 kHz. pClamp 9.2 software was used for data acquisition and analysis. The bath solution contained 145 mm NaCl, 5 mm CsCl, 1 mm MgCl_2_, 1 mm CaCl_2_, 10 mm Hepes, 10 mm glucose, and pH 7.4 (NaOH). Patch pipette resistance was between 3–5 megaohms after filling with pipette solution containing 150 mm cesium methane sulfonate, 8 mm NaCl, 10 mm Hepes, 10 mm EGTA, and pH 7.2 (CsOH). All recordings were carried out at RT.

### 2.9. Western Blot

Western blotting experiments were carried out according to our previous study [26, 27]. Briefly, cells were lysed in RIPA buffer (1% NP-40, 0.5% sodium deoxycholate, 0.1% SDS, 10 ng/mL PMSF, 0.03% aprotinin, and 1 *μ*M sodium orthovanadate) on ice for 30 min. Protein concentration of samples was measured with the bicinchoninic acid (BCA) assay method. Proteins were separated on 8–12% SDS-PAGE gels and transferred to nitrocellulose membrane (Millipore, USA). Membranes were blocked with 5% BSA in TBS with 0.1% tween-20 and incubated with primary antibodies as follows: anti-TRPM7 (1 : 1000, Abcam, #ab85016, USA), anti-p-Akt-Ser473 (1 : 1000, Cell Signaling Technology, #4060, Inc., USA), anti-Akt (1 : 1000, Cell Signaling Technology, #2920 Inc., USA), phospho-p44/42 MAPK (p-ERK1/2, 1 : 1000, Cell Signaling Technology, #8544, Inc., USA), anti-ERK1/2 (1 : 1000, Cell Signaling Technology, #4696, Inc., USA), and anti-*β*-actin (1 : 1000, Cell Signaling Technology, #3700, Inc., USA) antibodies followed by incubation with corresponding horseradish peroxidase-conjugated secondary antibodies were used against each primary antibody. Bands were developed with a chemiluminescence reagent system (Beyotime, China).

### 2.10. Statistical Analysis

Data are presented as means ± SEM. Two-way unpaired Student's* t*-test was used to compare the statistical significance between two groups, and ANOVA with subsequent Newman-Keuls test was used for multiple comparisons. *p* < 0.05 was considered statistically significant for all tests.

## 3. Results

### 3.1. Carvacrol Reduces TRPM7-Like Currents in PCa Cells

**Figure 1:**
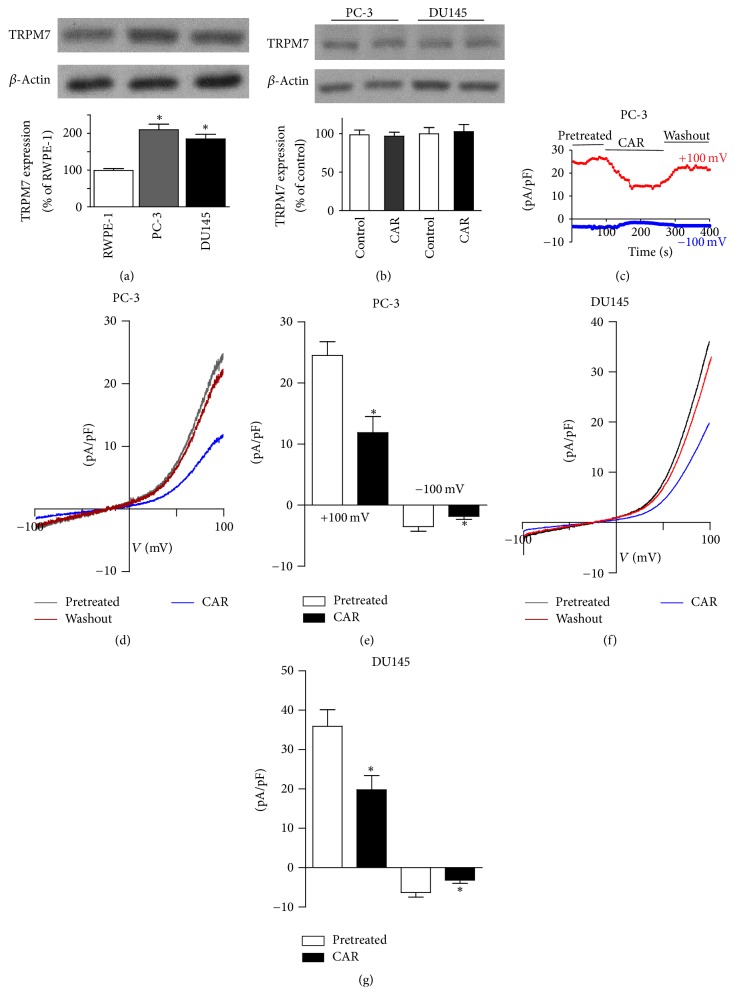
Carvacrol (CAR) inhibited TRPM7-like currents in PCa cells. (a) TRPM7 protein expression detected by western blotting (^*∗*^ *p* < 0.05 versus RWPE-1 cells, *n* = 6). (b) PC-3 and DU145 cells were treated with carvacrol (500 *μ*M) for 24 h. TRPM7 protein expression was detected by western blot. (c) Representative current traces of inward and outward currents at +100 mV and −100 mV (*n* = 3). The current traces were started to record when the TRPM7-like currents reached a platform after the finish of the whole cell configuration. Both inward and outward currents were inhibited by carvacrol (500 *μ*M), and they recovered after carvacrol washout. (d) Representative current-voltage trace of TRPM7-like current in PC-3 cells treated with either vehicle control (pretreated) or carvacrol (500 *μ*M). (e) Statistical analysis of current density at +100 mV and −100 mV in PC-3 cells (^*∗*^ *p* < 0.05 versus pretreated, *n* = 6). (f) Representative current-voltage trace of TRPM7-like current in DU145 cells treated with either vehicle control or carvacrol (500 *μ*M). (g) Statistical analysis of current density at +100 mV and −100 mV in DU145 cells (^*∗*^ *p* < 0.05 versus pretreated, *n* = 6).

We determined TRPM7 protein expression in RWPE-1, PC-3, and DU145 cells. As shown in Figure 1(a), western blotting results showed that TRPM7 protein expressed in these cells was higher in prostate cancer cell lines (PC-3 and DU145) than that in normal control prostate cell, RWPE-1. Carvacrol treatment for 24 h did not significantly affect TRPM7 expression of PC-3 and DU145 (Figure 1(b)). Next, we employed whole cell path-clamp to record TRPM7-like currents in PC-3 and DU145 cells. The current density in PC-3 and DU145 at +100 mV was 24.5 ± 2.3 pA/pF (Figures 1(c), 1(d), and 1(e)) and 35.9 ± 4.2 pA/pF (Figures 1(f) and 1(g)). Carvacrol (500 *μ*M) significantly reduced TRPM7-like currents (at +100 mV) in PC-3 and DU145 cell by ~52% and 45% (*p* < 0.05, *n* = 6), respectively. Besides, carvacrol (500 *μ*M) significantly reduced the currents at −100 mV in PC-3 and DU145 cells (Figures 1(e) and 1(g)).

### 3.2. Carvacrol Inhibits PC-3 and DU145 Cell Proliferation

**Figure 2:**
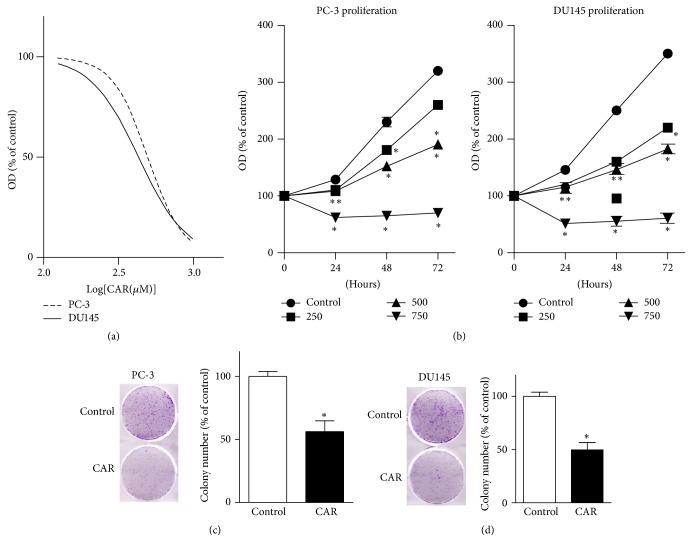
Carvacrol reduced PCa cell proliferation. (a) PC-3 and DU145 cells were treated with 125~1000 *μ*M for 24 h, then CCK-8 assay was conducted, and IC_50_ was calculated through nonlinear regression fitting (*n* = 6). IC_50_ in PC-3 and DU145 cells were 498.3 ± 12.2 *μ*M and 430.6 ± 21.9 *μ*M, respectively. OD is the abbreviation of optical density. (b) PC-3 cells (the left panel) and DU145 cells (the right panel) were treated with either vehicle control or carvacrol with 250, 500, and 750 *μ*M for 24, 48, and 72 hours, respectively. Then cell proliferation curves were detected by CCK-8 assay (^*∗*^ *p* < 0.05 versus control, *n* = 6). (c) Carvacrol reduced colony formation of PC-3 cells (^*∗*^ *p* < 0.05, *n* = 6). (d) Carvacrol reduced colony formation of DU145 cells (^*∗*^ *p* < 0.05, *n* = 6). *∗∗* refers to the comparisons of carvacrol at 250 and 500 *μ*M, respectively.

Then, we evaluated the effects of carvacrol on the proliferation of PCa cells. As shown in Figure 2(a), CCK-8 assay results showed that carvacrol reduced the viability of PC-3 and DU145 cells in a dose-dependent manner, with IC_50_ of 498.3 ± 12.2 *μ*M and 430.6 ± 21.9 *μ*M, respectively. As shown in Figure 2(b) (left panel), the proliferation of PC-3 cells in the control group increased with time (128.9 ± 3.0%, 230.1 ± 8.4%, and 320.1 ± 5.7% at 24, 48, and 72 hours). When cells were treated with 250, 500, and 750 *μ*M carvacrol, the rate of cell proliferation significantly decreased at 24, 48, and 72 hours (*p* < 0.05, *n* = 6). Meanwhile, we observed the similar effects of carvacrol on cell proliferation of DU145 (Figure 2(b), right panel). We further determined the antiproliferation effects of carvacrol using colony formation experiments. As shown in Figures 2(c) and 2(d), 500 *μ*M carvacrol significantly reduced colony numbers of PC-3 and DU145 by 56.2 ± 8.6% and 49.8 ± 6.7%, respectively (*p* < 0.05, *n* = 6).

### 3.3. Carvacrol Reduces PCa Cell Migration

**Figure 3:**
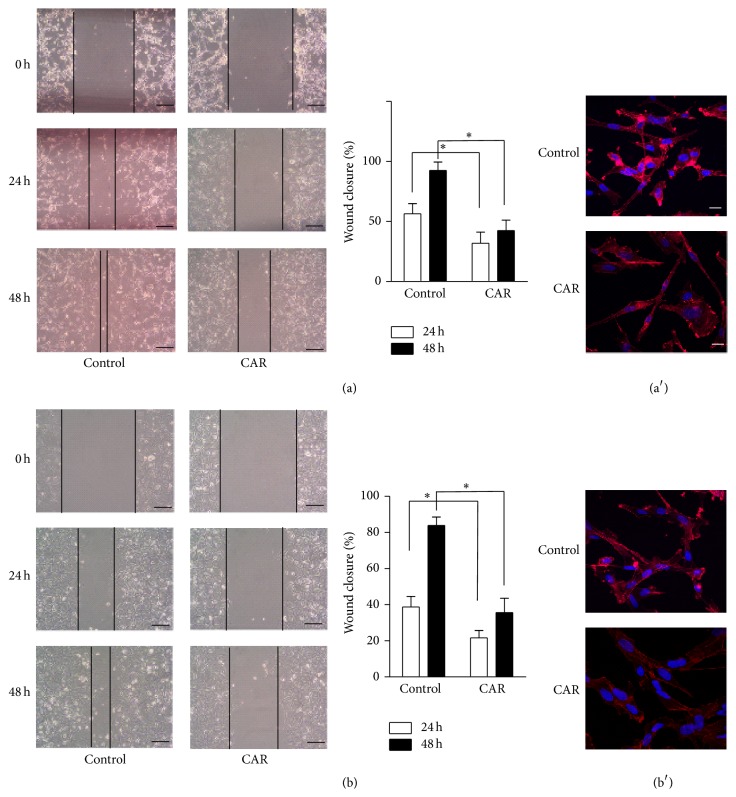
Carvacrol inhibited PCa cell migration. (a) PC-3 cells were treated with carvacrol (500 *μ*M) for 24 and 48 h. Images were taken at indicated time points. Wound closure was compared between vehicle control and carvacrol treated group (^*∗*^ *p* < 0.05, *n* = 6). (a′) Representative images of F-actin staining in PC-3 were showed (*n* = 6). (b) Wound healing assay and closure analysis were carried out as described in PC-3 cells (^*∗*^ *p* < 0.05, *n* = 6). (b′) Representative images of F-actin staining in DU145 cells were showed (*n* = 6).

Wound healing assay was carried out to detect cell migration. As shown in Figures 3(a) and 3(b), after treatment of 24 h, wound closures of PC-3 and DU145 in control group were 56.4 ± 8.5 and 38.7 ± 5.9, respectively. And after treatment of 48 h, wound closure of vehicle control in PC-3 and DU145 cells increased to 83.9 ± 4.2% and 92.5 ± 7.1%, respectively. Carvacrol significantly inhibited cell wound healing of PC-3 and DU145 cells (*p* < 0.05, *n* = 6), as the wound closure of PC-3 and DU145 cells in carvacrol treatment group was 31.8 ± 9.2 and 21.6 ± 4.1 at 24 h and 42.4 ± 8.6% and 35.6 ± 7.9% at 48 h, respectively. Thus, compared with vehicle control, carvacrol (500 *μ*M) significantly reduced PC-3 and DU145 cell migration.

As cell migration is related to reorganization of the actin cytoskeleton, we also measured the cytoskeletal actin organization by staining F-actin with phalloidin in PC-3 cells and DU145 cells. As shown in Figures 3(a′) and 3(b′), F-actin was condensed at the leading edge within structures resembling fans or protrusions in vehicle group. After treatment with carvacrol, less F-actin was condensed in dot-like structures at the margins of the cells, compared to vehicle control cells. The data suggest that inhibition of cell migration by carvacrol might be related to its prevention of F-actin reorganization.

### 3.4. Carvacrol Inhibits PCa Cell Invasion

**Figure 4:**
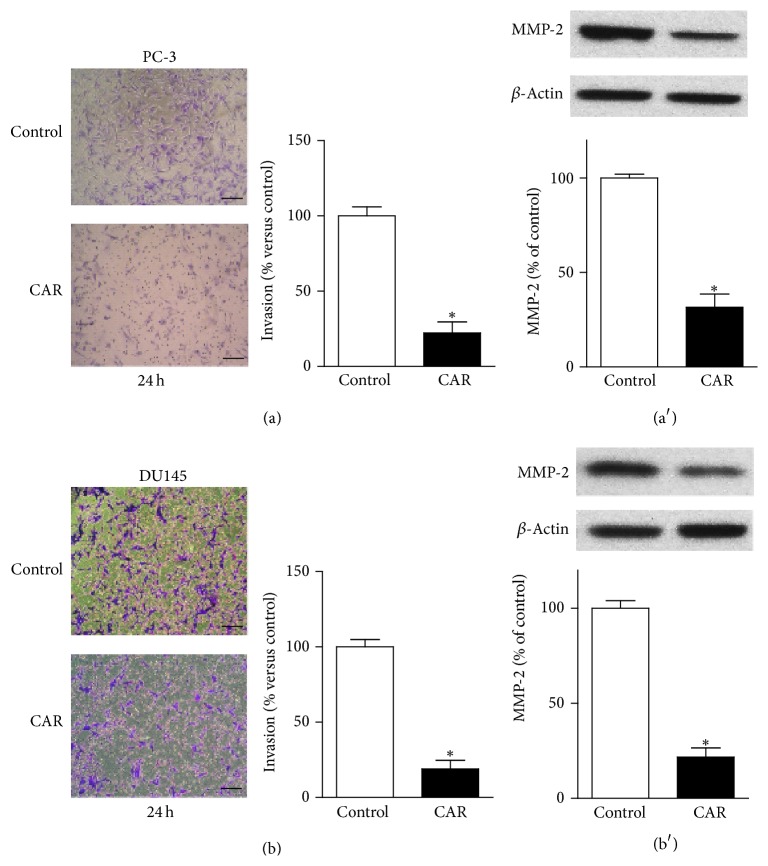
Carvacrol inhibited PCa cell invasion. (a) Representative images of Transwell assay in PC-3 cells were showed. Carvacrol treatment significantly reduced the invasion of PC-3 cells (^*∗*^ *p* < 0.05, *n* = 6). (a′) Western blot showed that carvacrol treatment for 24 h reduced MMP-2 protein expression in PC-3 cells (^*∗*^ *p* < 0.05, *n* = 6). (b) Representative images of Transwell assay in DU145 cells were showed. Carvacrol treatment significantly reduced the invasion of DU145 cells (^*∗*^ *p* < 0.05, *n* = 6). (b′) Western blot showed that carvacrol treatment for 24 h reduced MMP-2 protein expression in DU145 cells (^*∗*^ *p* < 0.05, *n* = 6).

We further detected whether carvacrol could inhibit PCa cell invasion using Transwell invasion assay. As shown in Figures 4(a) and 4(b), the results indicated that carvacrol (500 *μ*M) treatment significantly reduced PC-3 and DU145 cell invasion to 22.3 ± 7.2% and 18.9 ± 5.8% versus vehicle control, respectively (*p* < 0.05, *n* = 6). High expression level of MMP-2 suggests the strong ability of invasion. Thus, we determined MMP-2 expression using western blotting. As shown in Figures 4(a′) and 4(b′), western blotting results showed that carvacrol (500 *μ*M) treatment significantly reduced MMP-2 protein expression in both PC-3 and DU145 cells (*p* < 0.05, *n* = 6).

### 3.5. Carvacrol Suppresses PI3K/Akt and MAPK Signaling Pathways

**Figure 5:**
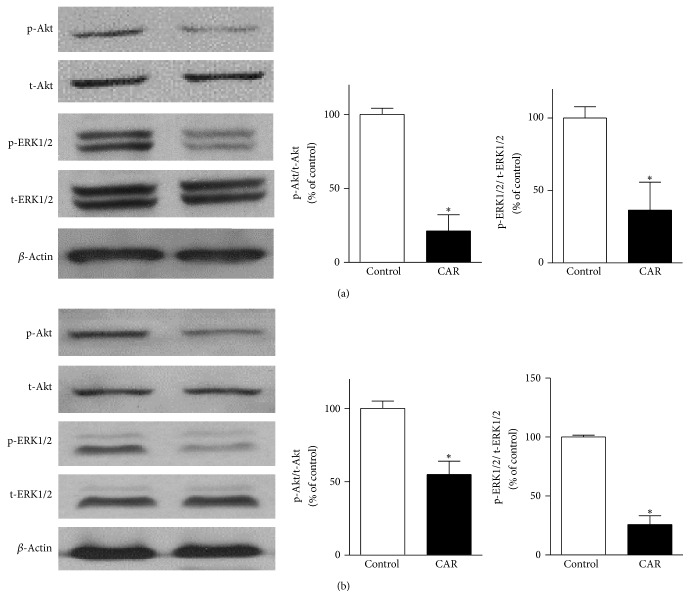
Carvacrol reduced p-Akt and p-ERK1/2 protein levels in PCa cells. (a) PC-3 cells were treated with carvacrol (500 *μ*M) for 24 h, and then western blotting experiments were carried out to detect the indicated protein expression (^*∗*^ *p* < 0.05, *n* = 6). (b) DU145 cells were treated with carvacrol (500 *μ*M) for 24 h, and then western blotting experiments were carried out to detect the indicated protein expression (^*∗*^ *p* < 0.05, *n* = 6).

Next, we studied the underlying signaling pathway involved in the anti-PCa effects of carvacrol. As shown in Figure 5, in carvacrol treatment group, phosphorylation of p-Akt and p-ERK in PC-3 cells was significantly reduced to 21.2 ± 4.5% and 36.4 ± 7.9% of vehicle control (*p* < 0.06, *n* = 6). In the meantime, similar results were observed in DU145 cells. The data suggest that PI3K/Akt and MAPK signaling pathways are involved in anti-PCa effects of carvacrol.

### 3.6. TRPM7 Knockdown Regulates the Functions of Prostate Cancer Cells

**Figure 6:**
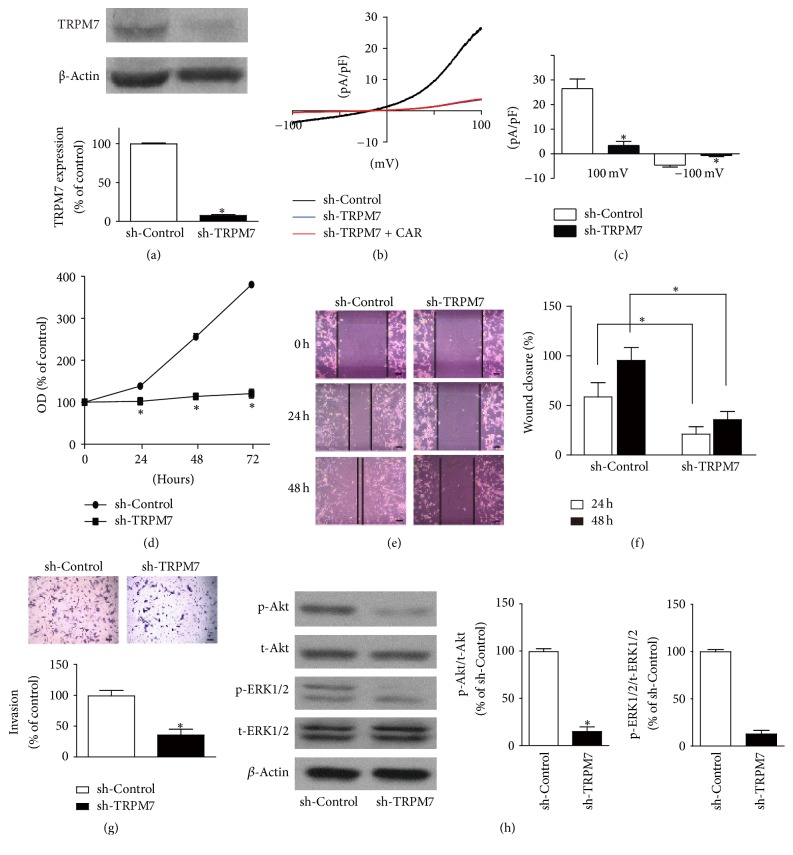
TRPM7 knockdown regulates PC-3 functions. (a) TRPM7 protein expression was determined by western blot after PC-3 infected with lentivirus vector (sh-Control and sh-TRPM7) for 72 h (^*∗*^ *p* < 0.05 versus sh-Control, *n* = 4). (b) After 72 h infection with either sh-Control or sh-TRPM7 lentivirus, TRPM7-like currents were recorded using patch clamp. Representative* I*-*V* traces of PC-3 cells were shown (*n* = 6). (c) Statistical analysis of patch clamp results was shown. TRPM7 knockdown significantly reduced the currents at +100 mV and −100 mV (^*∗*^ *p* < 0.05, *n* = 6). (d) TRPM7 knockdown significantly inhibited PC-3 cell proliferation detected by CCK-8 assay (^*∗*^ *p* < 0.05 versus sh-Control, *n* = 6). (e) TRPM7 knockdown significantly inhibited PC-3 cell migration detected by wound healing assay. The representative images were shown (*n* = 4). (f) Statistical analysis of wound healing (^*∗*^ *p* < 0.05, *n* = 4). (g) TRPM7 knockdown significantly inhibited PC-3 cell invasion detected by Transwell assay (^*∗*^ *p* < 0.05 versus sh-Control, *n* = 4). (h) TRPM7 knockdown significantly reduced p-Akt and p-ERK1/2 protein levels in PC-3 cells (^*∗*^ *p* < 0.05 versus sh-Control, *n* = 4).

As shown in Figure 6(a), PC-3 cells infected with lentivirus vector with shRNA-TRPM7 for 72 h significantly decreased TRPM7 protein expression, comparing with shRNA-Control (*p* < 0.05, *n* = 4). Moreover, TRPM7 knockdown significantly reduced TRPM7-like current as well (*p* < 0.05, Figures 6(b) and 6(c)), whereas carvacrol did not further significantly reduce TRPM7-like currents in TRPM7 knockdown PC-3 cells (Figure 6(b)). TRPM7 knockdown significantly inhibited PC-3 cell proliferation (Figure 6(d)), migration (Figures 6(e) and 6(f)), and invasion (Figure 6(g)). The protein levels of p-Akt and p-ERK1/2 were reduced by TRPM7 knockdown as well (Figure 6(h)).

## 4. Discussion

In the present study, we demonstrated that carvacrol inhibited TRPM7-like currents in PCa cells and reduced cell proliferation, migration, and invasion of PCa cell. Furthermore, we found that carvacrol treatment decreased MMP-2, p-Akt, and p-ERK protein expression and blocked F-actin reorganization in PCa cells. Consistently, TRPM7 knockdown inhibited PC-3 cell proliferation, migration, and invasion. It also suppressed p-Akt and p-ERK protein expression in PC-3 cells as well. TRPM7 channels are widely expressed in a variety of cells including prostate tissues [28]. Activation of TRPM7 promotes prostate cancer cell proliferation, migration, and viability [10, 11], while inhibition of TRPM7 by Gd^3+^ or 2-aminoethoxy diphenylborate (2-APB) enhances TRAIL-induced PC-3 cell apoptosis [12]. Carvacrol, an approved food flavor additive by the United States Food and Drug Administration (FDA), with oral LD_50_ is 810 mg/kg in rats [29] which was reported as a nonspecific TRPM7 inhibitor with IC_50_ of 306 ± 65 *μ*M [17]. Our data showed the inhibitory effects of carvacrol on TRPM7-like currents in PC-3 and DU145 cells, which was consistent with another researcher's study [7]. Furthermore, our results showed that both carvacrol treatment and TRPM7 knockdown significantly suppressed cell proliferation, migration, and invasion of PCa cells. These results suggest that blocking TRPM7 by carvacrol plays a key role in PCa growth and metastasis.

Cell adhesion and spreading properties directly regulate the cellular motility and invasiveness. F-actin dynamics is essential for alteration of cytoskeleton during cell migration and invasion [30]. We found that carvacrol treatment inhibited F-actin condensing at the leading edge of PCa cells, indicating that carvacrol reduced PCa cell motility through blocking F-actin-mediated cytoskeleton alteration. Matrix metalloproteinase-2 (MMP-2) is essential for focal extracellular matrix (ECM) degradation and invasion of the surrounding tissue. MMP-2 expression decreased in glioblastoma cells by treatment with carvacrol [7]. Consistently, we also found that carvacrol reduced MMP-2 protein expression in both PC-3 and DU145 cells. Hence, we could speculate that suppression of PCa functions by carvacrol might be closely related to regulation of F-actin and MMP-2 expression.

PI3K/Akt and MAPK signaling pathways are important in the PCa growth and metastasis [31, 32]. Phosphorylation of Akt and ERK is the key proteins regulating both signaling pathways, respectively. TRPM7 downexpression decreases the phosphorylation of p-Akt in ovarian cancer cells and lung fibroblasts and also decreases the phosphorylation of p-ERK1/2 in breast cancer cells [8, 33, 34]. In this study, our results showed that carvacrol reduced levels of p-Akt and p-ERK in both PC-3 and DU145 cells, which is consistent with another study [7].

Taken together, carvacrol treatment represses cell proliferation, migration, and invasion in both PC-3 and DU145 PCa cell lines, likely by blocking TRPM7-like current and reducing MMP-2 protein expression and F-actin dynamics. Moreover, both the PI3K/Akt and MEK/MAPK signaling pathways are involved in these antiprostate cancer effects. Our findings indicate that carvacrol has antiprostate cancer effects* in vitro*.
